# How Seasonality of Malnutrition Is Measured and Analyzed

**DOI:** 10.3390/ijerph18041828

**Published:** 2021-02-13

**Authors:** Anastasia Marshak, Aishwarya Venkat, Helen Young, Elena N. Naumova

**Affiliations:** 1 Feinstein International Center, Tufts University, Boston, MA 02111, USA; Helen.Young@Tufts.edu; 2 Friedman School of Nutrition Science and Policy, Tufts University, Boston, MA 02111, USA; Aishwarya.venkat@tufts.edu (A.V.); Elena.Naumova@tufts.edu (E.N.N.)

**Keywords:** acute malnutrition, seasonality, methodology, Africa, dryland

## Abstract

Seasonality is a critical source of vulnerability across most human activities and natural processes, including the underlying and immediate drivers of acute malnutrition. However, while there is general agreement that acute malnutrition is highly variable within and across years, the evidence base is limited, resulting in an overreliance on assumptions of seasonal peaks. We review the design and analysis of 24 studies exploring the seasonality of nutrition outcomes in Africa’s drylands, providing a summary of approaches and their advantages and disadvantages. Over half of the studies rely on two to four time points within the year and/or the inclusion of time as a categorical variable in the analysis. While such approaches simplify interpretation, they do not correspond to the climatic variability characteristic of drylands or the relationship between climatic variability and human activities. To better ground our understanding of the seasonality of acute malnutrition in a robust evidence base, we offer recommendations for study design and analysis, including drawing on participatory methods to identify community perceptions of seasonality, use of longitudinal data and panel analysis with approaches borrowed from the field of infectious diseases, and linking oscillations in nutrition data with climatic data.

## 1. Introduction

Seasonal variations occur in most human activities and natural processes, interconnected and part of the environmental and meteorological patterns of the ecosystems. The recognition of seasonality as a source of temporal variability is present across most disciplines, including ecology, anthropology, biology, food systems, and animal and human health. Less attention has been given to building the evidence base around the seasonality of malnutrition, despite (or maybe because of) the multi-causal nature of nutrition outcomes relating to almost all other disciplines. However, as we strain to meet the targets of the nutrition Sustainable Development Goals (SDGs) and acknowledge that, in some contexts, we are persistently seeing levels of malnutrition equated to a humanitarian emergency, despite the presence of an emergency [1], it is time to emphasize and better quantify the role of seasonality in nutrition outcomes.

Seasonality, a systematic periodic occurrence of events over the course of a year, is a well-known phenomenon in human health conditions manifested by marked fluctuations in the incidence of diseases [2]. The main determinants of temporal variations in health conditions are related to evolving host susceptibility, periodicity in exposure to pathogens, food availability, and the ever-changing environment that can support or repress a host’s growth and health. Seasonality in malnutrition refers to any pattern or variability in nutrition outcomes that are correlated with the seasons, specifically with changes in environmental conditions such as temperature, rainfall, and vegetation. Seasonal changes in these variables, mediated through livelihoods and institutions, underpin the underlying—food security, childcare, health—and immediate—disease and food consumption—drivers of child acute malnutrition [3]. Thus, the manifestations of acute malnutrition, most commonly measured by the weight for height z-score (WHZ), mid-upper arm circumference (MUAC), wasting defined as having a WHZ less than negative two standard deviations [4] or mid-upper MUAC less than or equal to 125 mm, and severe wasting defined as having WHZ less than negative three standard deviations or MUAC less than 110 mm, tend to exhibit a high level of variability over a short period of time [5,6,7,8,9,10]. Global acute malnutrition (GAM) refers to the prevalence of wasting in a population and severe acute malnutrition (SAM) refers to the prevalence of severe wasting (WHZ<-3). In addition, underweight, defined as having a weight for age z-score (WAZ) below negative two standard deviations, also partially captures acute malnutrition given its construction. The observed variability in acute malnutrition within the course of the year can be even greater than the variability observed across years [11]. However, the evidence base for seasonality is extremely limited, resulting in an overreliance on general assumptions. Thus, more recently, there has been a call for a better understanding of the seasonality of acute malnutrition, with seasonality identified as the “missing link” [6] or major gap in acute malnutrition prevention [1,3,12].

While seasonality of acute malnutrition is observed in many climatic conditions, it might be most pronounced in locations prone to environmental extremes. Drylands, defined as having an aridity index of less than 0.65 [13], are characterized by erratic rainfall, seasonally high temperature, and significant environmental variability across both time [1,13,14] and space [15]. Livelihood activities are ecologically adapted to the dryland environment, with communities taking advantage of the seasonal distribution of natural resources. Thus, the climatic variability and hence seasonality, mediated through livelihoods and institutions, are reflected in the immediate and underlying drivers of acute malnutrition, as well as the nutrition outcomes themselves. Populations living in drylands experience marked seasonal variability and stress across multiple outcomes, including child nutrition [16,17,18]. Many of the countries identified as drylands also correspond to humanitarian contexts that experience emergency levels of acute malnutrition, even in the absence of an emergency and significant humanitarian and development funding [19]. It is not surprising that over the last decade, international donors have spent roughly 90 billion dollars in just nine countries with large dryland areas, accounting for almost 50 percent of all humanitarian assistance worldwide [20]. Existing assumptions around malnutrition seasonality dictate the content and timing of programming, infrequently supported by evidence [21]. Thus, in this paper, we review the analytical approaches for existing nutrition seasonality research in drylands, as well as making recommendations on how to improve this research in order to design more appropriate interventions implemented at the correct time.

The three main features that characterize seasonality are: (1) a point in time when a seasonal curve reaches its maximum; (2) an amplitude from peak to nadir, and (3) a duration of a seasonal increase defined by a shape of a curve [2]. The shape of a seasonal pattern reflects how quickly a temporal curve reaches its peak and declines to a nadir over the course of a full cycle, typically a year or a half of a year. Therefore, the seasonal curve of a nutrition outcome can be characterized by periods of high or low malnutrition and could have one or more seasonal peaks. Quantifying these seasonal characteristics of acute malnutrition is critical for measuring progress towards the SDGs and the development of appropriately timed nutrition-sensitive and nutrition-specific interventions to reach those goals. More so, without a clear understanding of the seasonality of wasting, it is impossible to either interpret the aggregated global estimate of wasting [6] or report on trends in wasting, as is done with stunting and obesity [22].

Most existing seasonality studies in nutrition rely on categorical measures of the “time” variable, comparing nutrition outcomes across two to four time periods in the year. The selection of the timing of data collection is either based on implicit assumptions that acute malnutrition is seen to be caused first and foremost by a lack of food [21], by selecting seasons such as pre- vs. post-harvest, or using even-interval quarterly data collection. The categorical approach to “time” does not correspond to how time is analyzed in other public health fields that exhibit significant seasonal oscillation. Given that acute malnutrition is a symptom of disease [3,23], a cause of disease [24,25], and shows significant with-in year variability [11] similar to infectious diseases, we draw on analytical applications from the field of epidemiology that model and forecast oscillations in disease. Seasonality in the epidemiology literature is characterized by the magnitude, timing, and duration [26] of seasonal increases and thus requires sufficiently frequent observations, analyzed as a continuous variable, in order to capture those components and establish an understanding of seasonality. The use of a categorical time variable in both the design and analysis stage does not correspond to the reality of climatic variability, the relationship between that climatic variability and human activities, and instead might inadvertently support existing assumptions instead of contributing to a robust evidence base.

The aim of this paper is to review the existing methods and models for assessing seasonality in acute malnutrition. We then proceed to offer recommendations for primary data collection, study design, and data analysis. Throughout the review, we specifically narrow in on dryland given the high level of seasonal variability and possible comparability given the similar environmental context. However, an understanding of the seasonality of acute malnutrition is critical beyond just the dryland context and is necessary for designing appropriately timed interventions and tracking progress towards the SDG goals more broadly.

## 2. Materials and Methods

We carried out a scoping review to identify the literature on the seasonality of acute malnutrition in drylands. We based our search on the following inclusion criteria:

- Objective: While the article did not have to list seasonality analysis as an objective, some measure of seasonality had to be included in the analysis, either modeled or visually.
- Geography: Only articles looking at drylands were evaluated. These included the following countries: Benin, Botswana, Burkina Faso, Chad, Ethiopia, Eritrea, Gambia, Ghana, Kenya, Mali, Malawi, Namibia, Niger, Nigeria, South Africa, South Sudan, Sudan, Zimbabwe in Africa; Chile in South America; and Pakistan, Mongolia, Kazakhstan, and Afghanistan in Asia. Countries were identified as drylands according to whether they had territory with an aridity index less than 0.65 [27,28].
- Outcome measures: Only articles that included a measure of acute malnutrition, either wasting, severe wasting WHZ, MUAC, global acute malnutrition (GAM), and/or severe acute malnutrition (SAM), as well as weight for age (WAZ) and underweight (WAZ < −2) given the inclusion of weight in the construction.

However, information on modeling approaches, such as seasonal analysis, is notoriously difficult to extract through regular search engines. For example, we did a scrape on Google Scholar on the search terms “seasonal”, “child”, and “acute malnutrition”, and all their possible variations, as well as the geographical criteria. We specified that the search terms must appear in either the title, abstract, or keywords. We set the start date to 1920. This approach allowed us to identify 548 articles. We then applied our “outcome” criteria to the article as well as confirming that some findings on seasonality were presented or included in the models, which reduced the number of articles to 9. More importantly the search missed 15 of the articles identified through non-systematic approaches, such as communication with other researchers and reviewing bibliographies. Combing all approaches, we identified 24 articles that met all three criteria upon careful review. Notably, no research on seasonality of nutrition outcomes in dryland contexts was found for either South America or Asia, despite multiple countries in the latter being identified as having persistently high levels of acute malnutrition [19].

## 3. Results

Seasonality has been a critical component of research and programming around agriculture [18] and infectious diseases [26,29,30,31,32,33], but less so when it comes to nutrition. However, there is a growing body of evidence that aims to quantify the seasonality of acute malnutrition in drylands with a mix of different design and analytical approaches. In this section, we present a summary of those methodological approaches and their respective advantages and disadvantages in measuring and analyzing nutrition seasonality in Africa’s drylands.

Table 1 provides a summary of temporal measures, data types, e.g., cross-sectional vs. longitudinal, statistical tests used to perform seasonality analysis, and nutrition variable under study, as presented in the reviewed literature related to nutrition seasonality. Over half of the studies (13) reviewed compare across two to four time periods, with either a month selected to be representative of a predefined season, aggregation of data across several months to create a seasonal mean, or the use of even-interval quarterly data. There is a mix of both cross-sectional and longitudinal studies, but a similarity across statistical methods. The analytical approaches utilized in these studies to establish seasonality are primarily the comparison of the time periods using paired and unpaired *t*-tests (4), multivariate analysis with the inclusion of season as a dummy variable (5), and/or repeated-measure analysis of variance (3). In addition, six papers drew conclusions from visual representations of nutrition outcomes by month; two papers used harmonic regression; three papers used random-, fixed-, or mixed-effects analysis; one paper used Bayesian hierarchical space–time modeling, and one paper used Fourier regression.

Each analytical approach is associated with distinct benefits, but also caveats, as presented in Table 2. A clearer understanding of the options for analysis can help to identify opportunities and appropriate study designs. The use of *t-*tests is the simplest form of analysis when comparing nutrition outcomes across two to four seasonal categories. The test itself is well understood and the results easy to interpret. Complexity slightly increases when running multivariate analysis with the inclusion of the season as an indicator, or dummy variable, but still allows for easy interpretation and for controlling for other key characteristics, such as the age and sex of the child. With only two to four seasons and hence a maximum of three variables indicating a season, multivariate analysis does not require a long time series of data that define a sample size needed for the analysis. These approaches can be applied to both cross-sectional and longitudinal data. Repeated-measures analysis of variance can further improve the analysis by taking advantage of the panel nature of the data and hence controlling for factors that cause variability between subjects. However, because all three of these analytical approaches require “time” to be included as a categorical variable, they force several assumptions on the model that do not correspond to the environmental variability characteristic of drylands and violate basic principles of time continuity.

The categorical classification of seasons is not appropriate for dryland contexts as seasons are not fixed with respect to calendar months. There can be significant variability with respect to the timing of the rains in a given location and hence when the “rainy” season happens versus the “dry” season. For example, in the Ouaddai Region of Chad, the beginning of the rains can typically start as early as March or as late as June and end as early as August or as late as December [1]. Thus, having a fixed month to serve as representative of that season could be capturing two very different climatic conditions and hence the presence of different underlying and immediate drivers of acute malnutrition. Furthermore, the “rainy” periods vary in duration and intensity over time and across locations.

Similarly, taking a mean of multiple months to represent a seasonal mean does not take account of the climatic variability in drylands. A few studies aggregated nutrition data across pre-determined seasons, thus ultimately reducing the precision of the data. The problem with this approach is that a lot of information can be lost in the aggregation of seasonal data [29,34], especially if the critical period for peak wasting is likely to be short [7], as is the case in the findings around the start of the rains being a critical period of acute malnutrition risk in Chad, Sudan, and South Sudan [1].

The pre-selection of two to four seasons also does not correspond to community perceptions of seasonality in drylands. For example, among the Fulani, three or four major seasons are recognized, but communities also identify sub-seasons that relate directly to their annual cycle of agricultural and pastoral activities, reporting up to eight seasons [35]. In Chad and Sudan, households identify five seasons, which include categories that reflect the start of the rains and period between the harvest and dry season, on top of the more standard categories of rainy, dry, and harvest season [1,36,37]. These periods of transition between the standard seasonal categories can be fairly short, up to three weeks, but are extremely vital. Recently, several studies have identified the start of the rains, an extremely short season lasting roughly three weeks, as the period of greatest risk of acute malnutrition in Africa’s drylands [1].

Furthermore, the pre-selection of specific seasons and their identification as “harvest”, “pre-harvest”, and “post-harvest” or “moderate hunger”, “hunger”, and “post hunger” implies a built-in assumption of food security or production yield as the primary driver of acute malnutrition, corresponding to the food-first bias [21]. While the use of even-interval selection of timing of data collection decreases the possibility of built-in assumptions around specific seasons, there is a clear implication here, likely borrowed from western culture, that seasons are equal in duration. However, seasons as described by local populations follow the pattern of human activities and access to specific natural resources and thus almost never fall into evenly spaced intervals.

A handful of studies (4), on the other hand, utilized random-, fixed-, or mixed-effects modeling, treating time as a continuous variable and thus avoiding some of the built-in assumptions present with categorical data. Two of these studies applied harmonic regression analysis typical of epidemiology and used in the modeling of infectious diseases [26,31,38]. However, both studies limited the model to the inclusion of 2π sine and cosine terms that refer to a single seasonal peak and thus could not accommodate a more complex form in seasonal variations relevant to food security concepts. The exclusion of higher-order harmonic terms means that the model directly embedded the assumption of just one peak in the nutrition outcome, which might not be the case in all drylands [1].

## 4. Discussion

Given the importance of environmental variability and seasonality in relation to peak timing of acute malnutrition and its drivers [3], there are several approaches to improving both the design and analysis of nutrition seasonality research, with an emphasis on borrowing from techniques developed and tested in modeling and forecasting infectious diseases, given the clear link with disease as both a cause and effect of acute malnutrition and similar within-year oscillation. Furthermore, analytical methods in the field of epidemiology have advanced and adapted to more complex and developmental settings, characteristics of the context that have significant malnutrition [39,40]. A better understanding of these techniques can not only improve the accuracy of the analysis, but also better inform nutrition seasonality study designs, as well as provide improved options for analysis of data originally collected with a categorical measure of seasonality. We provide several recommendations below with an emphasis on incorporating participatory methods to capture household perspectives on seasonality, lining up the quantitative data collection and analysis with climatic variability by drawing on remote sensing data, opportunities for drawing on secondary data for prediction of seasonal peaks in acute malnutrition, and general tips for improving the precision of seasonality analysis.

People’s livelihoods, such as pastoralism, farming, and fishing, require access to natural resources, access that is directly related to environmental variability, particularly in dryland contexts. The ways in which these resources are used, mediated through institutions and social norms, influence the immediate and underlying drivers of acute malnutrition. Thus, greater value must be placed on indigenous knowledge and local perspectives on seasonality. Communities distinguish between multiple seasons, which is extremely useful for the anticipation and description of environmental changes and human activities [7,35] that need to be taken into account in seasonality research alongside more quantitative methods. Without the investment in participatory methods to identify the seasons as they relate to household livelihoods and activities, there is a danger of accidentally missing a critical season when it comes to both research and programming design.

In addition to a strong investment in participatory methods, to guide a seasonal understanding of the context, quantitative methods need to be in line with seasonal variability. While collection of high frequency longitudinal data should be prioritized as it allows for the use of mixed-effects modeling with the harmonic terms (Table 2), the use of remote sensing data can help to align changes in nutrition outcomes with climatic variability. As discussed above, dryland contexts exhibit significant variability and thus calendar months cannot be consistently associated with a specific season. For example, in Chad, data collection in June might capture the dry season, the initial intermittent rains, or the rainy season, all depending on the year of data collection [1]. Thus, seasonal nutrition variability can be best understood when analyzed with respect to variables such as precipitation, temperature, and vegetation, which was done by a few of the nutrition seasonality studies [1,11].

Approaches to incorporating climatic data for better acute malnutrition modeling and forecasting can be borrowed from the existing infectious disease literature [29,30]. For example, our recent study dealing with the complex non-linear structure with strong seasonality of rotaviral infections introduced a novel two-step procedure to estimate seasonal features and understand the relationship between infection and environmental conditions for the purpose of peak timing forecasting [41]. Another study used agglomerative clustering methods to create a composite of meteorological conditions or rule of thumb to detect days favoring salmonellosis outbreaks in a given location [42]. In this approach, we are taking advantage of a data-driven methodology to navigate how to create “seasons” which are meaningful in a specific context.

There are also opportunities to take advantage of existing secondary data, applying some of the analytical techniques used on high frequency longitudinal data (Table 2). Many of the dryland contexts in Africa also experience frequent humanitarian emergencies and therefore entail significant and consistent data collection of nutrition outcomes. Taking advantage of all available raw survey data across multiple surveys, rather than just an individual survey, can provide many observations across time, allowing for the enumeration area to serve as the unit of analysis and hence turning cross-sectional data into longitudinal data. For example, Chotard et al. take advantage of 900 SMART surveys carried out across the Horn of Africa to examine seasonal trends [43]. Nielson et al. used 164 publicly available surveys carried out in the Darfur region of Sudan to carry out seasonality analysis, utilizing the day of survey collection to create a continuous time variable for analysis [44]. Another analysis looked at 300 SMART surveys over 20 years in Chad, Sudan, and South Sudan to identify two seasonal peaks of acute malnutrition [1]. Finally, Thiede et al. utilized 39 Demographic and Health Surveys (DHS) across 18 countries over 25 years [45].

When it comes to the analysis of seasonality features, there are multiple ways to improve data quality and to increase the precision and depth of data analysis irrespective of the design stage. Information on the day of data collection, instead of a rough recording of a month, even if the study uses a monthly or quarterly design, should be widely utilized and strongly enforced for analysis purposes [44]. Given that most data are now collected on digital data collection devices (i.e., tablets, smart phones, etc.), information such as day and time of data collection could be included automatically as a timestamp and in the most efficient and appropriate format, including year, month, and day, to allow efficient data sorting, arranging, and verification. With improved temporal granularity data, analysts could expand the range of models and convert time units into the most suitable aggregation, such as a week or a month, if needed [29].

In addition, the collection of global position systems (GPS) coordinates is also frequently automatic when conducting digital data collection. Inclusion of this information can allow for easier and proper linkages with remote sensing data, thus allowing the direct analysis of nutrition outcomes with respect to changes in rainfall, vegetation, temperature, as well as land use, topology, proximity to markets, health centers, etc., as opposed to using a crude assignment of a season or a rough proxy as a month indicator in an analytical model to deal with the temporal and spatial heterogeneity.

At the modeling stage, seasonality analysis needs to check for multiple peaks in nutrition outcomes by utilizing harmonic regressions with 2π, 4π, and higher-order sine and cosine terms or using non-parametric data-driven methods. Recently, several studies have identified more than one seasonal peak in acute malnutrition in unimodal dryland contexts [1]. It is possible that even more peaks exist in more complex climatic conditions, such as in bimodal rainfall drylands. All analysis also needs to occur across both the binary (wasting) and continuous form (WHZ) of acute malnutrition given that the two forms capture different information (tail end of the distribution versus median) and thus reflect different programmatic implications. In addition, data need to be explored separately for boys and girls, as they might have different drivers and seasonal trends in nutrition outcomes, thus requiring different programmatic approaches. Multiple studies have found boys to have significantly worse nutrition outcomes compared to girls [46,47,48,49,50,51], as well as different seasonal patterns [50,52]. Thus, an investment in sex-stratified seasonality research is critical in order to design appropriate interventions.

Modeling seasonality in the fields of infectious disease epidemiology could provide a rich ground for methodology cross-fertilization. Not only is such research valuable for a deeper understanding of the relationship between malnutrition and infections, but the methodological aspects and data sources could increase the range of available approaches. For example, in our study in India, we used systematically collected hospitalization records, taking advantage of detailed patients place of origin data, to provide insight into the seasonality of cholera at the point of care to support comprehensive national infectious disease surveillance [39]. This approach is relevant for countries where the nutrition outcomes are routinely collected at local clinics, health centers, and hospitals. Research from Costa Rica used hospital admission data to estimate peak timing of rotavirus infections and to provide the benchmark at the start of the national vaccination campaign [40]. Such use of centralized records could be of value for monitoring the impact of intervention strategies. Existing surveillance data can be also used for both forecasting purposes as well as providing insight into historical outbreaks. Our study in Australia used surveillance data on Ross River virus and Barmah Forest virus to identify peak timing, as well as to estimate the impact of a false-positive Barmah Forest virus epidemic in 2013 [53]. We had recently demonstrated the summertime synchronization of foodborne outbreaks in the USA using the national surveillance data [54]. Thus, secondary data could be extremely useful as part of formative research, for extrapolating seasonal trends from smaller and context specific primary seasonality research, as well as for general forecasting and modeling. Obviously, the analysis provided in this paper can be expanded beyond malnutrition in drylands and cover broader health and climatic aspects in future research.

With the recent explosion of open data sources, novel analytical tools, increased computation and visualization capacities, and broad interests in seasonal variations of health conditions, new opportunities are emerging to better understand the nature, drivers, and implications of interventions to improve nutrition and health outcomes. Seasonal patterns in malnutrition have been long recognized by epidemiologists and their links to environment and sensitivity to climatic fluctuations deserve close attention. Better assessment of seasonality, its characterization, analysis, and description is critical for health practitioners, researchers, and decision-makers for targeting the timing of interventions in a more accurate manner, for efficient allocation of precious resources, and for careful programming of preventive strategies geared toward nutrition and meeting the targets of the nutrition SDGs.

## 5. Conclusions

Understanding the seasonality of acute malnutrition is critical for designing appropriately timed interventions for both treatment and prevention, as well as tracking progress towards the SDG goals. Unfortunately, at the time of writing, the evidence base on the seasonal patterns of nutrition outcomes in Africa’s drylands is limited. However, there are significant opportunities to improve the design and analysis of the seasonality of acute malnutrition, borrowing from lessons learned and methods developed in the field of epidemiology. A better understanding of the benefits and caveats of different analytical methods can inform study design and improve analysis of seasonality more broadly. Child malnutrition is a complex phenomenon that requires us to draw across multiple fields of study: agricultural production, infectious diseases, nutrient content, water systems, cultural norms, livelihoods, gender, etc. Thus, our methods toolbox should be equally diverse, constantly learning from best practices and adapting to contextual constraints.

## Figures and Tables

**Table 1:** Summary of temporal measures, data types, statistical tests, and seasonal nutrition variables presented in the reviewed literature.

Temporal Resolution	Country	Temporal Measure	Data Type:	Statistical Tests	Nutrition Variable
Two time periods	Ethiopia	wet vs. dry season	Longitudinal	paired *t*-test	Wasting
	Chad	end of dry season vs. end of rainy season	cross- sectional	multivariate logistic regression	GAM
	Malawi	lean cropping season vs. post-harvest season	cross-sectional	multivariate logistic regression	Underweight
	Ethiopia	monthly, but grouped into post-harvest vs. pre-harvest	Longitudinal	random-effects regression	WHZ
	Kenya	dry vs. rainy	Longitudinal	mixed logistic regression model	SAM with complications
Three time periods	Mali	monthly, but grouped into harvest, dry, and rainy season	Longitudinal	repeated-measures analysis of variance	WHZ
	Greater Horn of Africa	secondary data grouped into moderate, hunger, and post-hunger season	cross- sectional	multivariate analysis	Wasting
Four time periods	Kenya	Aug-Nov, Dec-Feb, Mar-May, and Jun-Sep	Longitudinal	visual inspection	WHZ
	Gambia	May, October, February, September	Longitudinal	visual inspection	WHZ
	Senegal	February, June, October, December	Longitudinal	*t*-test, analysis of variance	WHZ
	Niger	November, February, May, September	Longitudinal	*t*-test and multivariate regression	Wasting
	Zimbabwe	four seasons: January-March, April-June, July-September, October-December	cross- sectional	multivariate regression	Under- weight
	Somalia	harsh dry season, rainy season, second dry season, short rainy season	cross- sectional	Bayesian hierarchical space-time model	Wasting
Monthly	Niger	monthly	cross- sectional	none, visual inspection	Severe wasting
	Malawi	monthly	Longitudinal	none, visual inspection	Wasting
	Chad, Sudan, South Sudan	monthly	cross- sectional	none, visual inspection	Wasting, WHZ
	South Sudan	monthly	cross- sectional	none, visual inspection	Wasting
	Malawi	monthly	cross-sectional	linear regression	# of children underweight
	Ghana	monthly	Longitudinal	*t*-tests, linear regression	Wasting, WHZ
	Sub-Saharan Africa	monthly	cross-sectional	fixed-effects regression	Weight, Wasting
	Kenya	monthly	Longitudinal	repeated-measures analysis of variance	MUAC for age
	Gambia	monthly	Longitudinal	Fourier regresson	WHZ
>Monthly	Nigeria	every 2–4 weeks	cross- sectional	mixed-effects harmonic regression with 2π terms	GAM
	Sudan	day of survey	cross- sectional	mixed-effects harmonic regression with 2π terms	GAM, SAM

GAM: Global acute malnutrition; WHZ: weight for height z-score; SAM: severe acute malnutrition; MUAC: mid-upper arm circumference.

**Table 2:** Summary of temporal resolution, analytical methods, and their respective benefits and caveats in analyzing nutrition seasonality.

Temporal Resolution	Analytical Method	Benefits	Caveats
Categorical variable representing seasons (e.g., wet vs. dry, pre-harvest vs. post-harvest)	*t*-test (paired, unpaired)	Easy to calculate and interpret	Assumes independent samples of two populations are normally distributed with same variance Cannot control for differences between populations
	Multivariate Regression	Can control for wide variety of covariates Variables can be transformed to meet assumptions Easy to interpret coefficients	Assumptions: Relationship between independent and dependent variables is linear All variables are multivariate normal Independent errors: no autocorrelation Homoscedastic errors: constant variance of errors, no multicollinearity Results can only be interpreted in comparison to reference category
	Fixed-Effect Regression	Useful for longitudinal data to control for subject-specific means Easy to interpret coefficients	Assumes unobservable factors influencing both independent and dependent variables are time-invariant Assumes constant group means, thus selection of grouping variables is critical Regression assumptions hold (see Multivariate Regression above)
	Bayesian Models	Flexible probability-driven method Does not rely on traditional regression assumptions	Requires selection of appropriate distributions to characterize prior and posterior beliefs Requires advanced numerical approximation algorithms (e.g., Laplace Approximation, Markov Chain Monte Carlo) Not widely utilized in development community
Categorical variables representing monthas a proxy to a season	Multivariate Regression	(see Multivariate Regression above) Use of indicator or dummy variables to represent temporal variable (as month or week) can obscure temporal sequence	
	Repeated-Measures ANCOVA	Useful for longitudinal data with large sample size and regular observations	Assumes population variances of all possible difference scores are equal (sphericity) Results can only be interpreted over fixed time points; must be serialized for continuous interpretation
Continious variable representing seasons in time units, e.g., month, week, or day	Harmonic Regression	Useful for cyclical phenomena Easy estimation of harmonic characteristics such as peak timing and amplitude	Begin modeling with more than one periodic term (2π, 4π, 6π, etc.) to account for possibility of more than one seasonal peak
	Classic and Modern Time Series Model	Useful for cyclical phenomena	Might be complex in interpretation and require specialized software
	Bayesian Models	(see Baysian Models above)	

## References

[1] FAO and Tufts (2019). Twin Peaks: The Seasonality of Acute Malnutrition, Conflict, and Environmental Factors in Chad, South. Sudan, and the Sudan.

[2] Naumova E.N. (2006). Mystery of seasonality: Getting the rhythm of nature. J. Publ. Health Policy.

[3] Young H. (2020). Nutrition in Africa’s Drylands: A Conceptual Framework for Addressing Acute Malnutrition.

[4] WHO, WHO Multicentre Growth Reference Study Group: WHO Child (2006). Growth Standards: Length/height-For-Age, Weight-For-Age, Weight-For-Lenght, Weight-For-Height and Body Mass Index for Age: Methods and Development.

[5] ACF (2013). Seasonality. The Missing Piece of the Undernutrition Puzzle?.

[6] Baye K., Hirvonen K. (2020). Seasonality: A missing link in preventing undernutrition. Lancet Child Adolesc. Health.

[7] Chambers R. (1983). Seasonality, Poverty, and Nutrition: A Professional Frontier, in EFNAG NAtional Workshop on Poverty and Malnutrition.

[8] Devereux S., Sabates-Wheeler R., Longhurst R. (2012). Seasonality, Rural Livelihoods and Development.

[9] Ulijasek S.J., Strickland S.S. (1993). Seasonality and Human Ecology.

[10] Valverde V., Delgado H., Martorell R., Belizán J.M., Mejia-Pivaral V., Klein R.E. (1982). Seasonality and nutritional status. A review of findings from developed and developing countries. Arch. Latinoam. Nutr..

[11] Kinyoki D.K., Berkley J.A., Moloney G.M., Odundo E.O., Kandala N.B., Noor A.M. (2016). Space-time mapping of wasting among children under the age of five years in Somalia from 2007 to 2010. Spat Spatiotemporal. Epidemiol..

[12] Wells J.C.K., Briend A., Boyd E.M., Berkley J.A., Hall A., Isanaka S., Webb P., Khara T., Dolan C. (2019). Beyond wasted and stunted—A major shift to fight child undernutrition. Lancet Child Adolesc. Health.

[13] Kratli S., Jode H. (2015). Valuing variability. New Perspectives on Climate Resilient Dyrlands Development.

[14] Kratli S., Sougnabe P., Staro F., Young H. (2018). Pastoral Systems in Dar Sila, Chad: A Background Paper for Concern Worldwide.

[15] Hutchinson C., Hermann S. (2008). The Future of Arid Lands-Revisited: A Review of 50 Years of Drylands Research.

[16] Foeken D., Hartog A. (1990). Seasons, Food Supply and Nutrition in Africa.

[17] Schofield S. (1974). Seasonal factors affecting nutrition in different age groups and especially preschool children. J. Develop. Stud..

[18] Chambers R., Longhurst R., Pacey A. (1981). Seasonal Dimensions of Poverty.

[19] Young H., Marshak A. (2017). Persistent Global Acute Malnutrition.

[20] USAID Resilience. https://www.usaid.gov/east-africa-regional/resilience.

[21] Pelletier D., Deneke K., Kidane Y., Haile B., Negussie F. (1995). The food-first bias and nutrition policy: Lessons from Ethiopia. Food Policy.

[22] WHO, UNICEF, World Bank (2020). Levels and Trends in Child. Malnutrition. Joint Child. Malnutrition Estimates (Key Findings of the 2020 Edition).

[23] UNICEF (1990). Strategy for improved Nutrition of Children and Women in Developing Countries. A UNICEF Policy Review.

[24] Brown K. (2003). Diarrhea and malnutrition. J. Nutr..

[25] Young H., Jaspers S. (1995). Nutrition, disease, and death in times of famine. Disasters.

[26] Ramanathan K., Thenmozhi M., George S., Anandan S., Veerarghavan B., Naumova E.N., Jeyaseelan L. (2020). Assessing seasonality variation with harmonic regression: Accommodations for sharp peaks. Int. J. Environ. Res. Publ. Health.

[27] Kolding J., Zwieten P., Marttin F., Poulain F., FAO (2016). Fisheries in the Drylands of Sub-Saharan Africa.

[28] Carvigini R., Morris M. (2016). Confronting drought in Africa’s Drylands: Opportunities for Enhancing Resilience, in Africa Development Forum Series.

[29] Alarcon Falconi T.M., Estrella B., Sempertegui F., Naumova E.N. (2020). Effects of data aggregation on time series analysis of seasonal infections. Int. J. Environ. Res. Public Health.

[30] Jagai J.S., Castronovo D., Monchak J., Naumova E.N. (2009). Seasonality of cryptosporidiosis: A meta-analysis approach. Environ. Res..

[31] Jagai J.S., Sarkar R., Castronovo D., Kattula D., McEntee J., Ward H., Kang G., Naumova E.N. (2012). Seasonality of rotavirus in South. Asia: A meta-analysis approach assessing associations with temperature, precipitation, and vegetation index. PLoS ONE.

[32] Naumova E.N., Christodouleas J., Hunter P., Syed Q. (2005). Effect of precipitation on seasonal variability in cryptosporidiosis recorded by the North. West. England surveillance system in 1990–1999. J. Water Health.

[33] Naumova E.N., Jagai J.S., Matyas B., DeMaria A., MacNeil I.B., Giffiths J.K. (2007). Seasonality in six enterically transmitted diseases and ambient temperature. Epidemiol. Infect..

[34] Fisman D.N. (2007). Seasonality of infectious diseases. Annu. Rev. Public Health.

[35] Hill A. (1985). Population, Health and Nutrition in the Sahel.

[36] Young H., Ismail M.A. (2019). Complexity, continuity and change: Livelihood resilience in the Darfur region of Sudan. Disasters.

[37] Saverio K., Eldirani O.H., Young H. (2013). Standting Wealth: Pastoralists Livestock Production and Local Livelihoods in Sudan.

[38] Sarkar R., Kang G., Naumova E.N. (2013). Rotavirus seasonality and age effects in a birth cohort study of southern India. PLoS ONE.

[39] Venkat A., Alrocon Falconi T.M., Cruz M., Hartwick M., Anandan S., Kumar N., Ward H., Veerarghavan B., Naumova E.N. (2019). Spatiotemporal patterns of cholera hospitalization in Vellore, India. Int. J. Environ. Res. Public Health.

[40] Urena-Castro K., Avila S., Gutierrez M., Naumova E.N., Ulloa-Gutierrez R., Mora-Guevara A. (2019). Seasonality of rotavirus hospitalizations at Costa Rica’s National Children’s Hospital in 2010–2015. Int. J. Environ. Res. Public Health.

[41] Alsova O.K., Loktev V.B., Naumova E.N. (2019). Rotavirus seasonality: An application of singular spectrum analysis and polyharmonic modeling. Int. J. Environ. Res. Public Health.

[42] Stashevsky P.S., Yakovina I.N., Alarcon Falconi T.M., Naumova E.N. (2019). Agglomerative clustering of enteric infections and weather parameters to identify seasonal outbreaks in cold climates. Int. J. Environ. Res. Public Health.

[43] Chotard S., Mason J.B., Oliphant N.P., Mebrahtu S., Hailey P. (2011). Fluctuations in wasting in vulnerable child populations in the Greater Horn of Africa. Food Nutr. Bull..

[44] Nielsen J., Prudhon C., de Radigues X. (2011). Trends in malnutrition and mortality in Darfur, Sudan, between 2004 and 2008: A meta-analysis of publicly available surveys. Int. J. Epidemiol..

[45] Thiede B.C., Strube J. (2020). Climate variability and child nutrition: Findings from sub-Saharan Africa. Global Environ. Change.

[46] Svedberg P. (1990). Undernutrition in Sub-Saharan Africa: Is there a gender bias?. J. Develop. Stud..

[47] Black R.E., Victora C.G., Walker S.P., Bhutta Z.A., Christian P., De Onis M., Ezzati M., Grantham-McGregor S., Katz J., Martorell R. (2013). Maternal and child undernutrition and overweight in low-income and middle-income countries. Lancet.

[48] Wamani H., Astrom A.N., Peterson S., Tumwine J., Tylleskar T. (2007). Boys are more stunted than girls in sub-Saharan Africa: A meta-analysis of 16 demographic and health surveys. BMC Pediatr..

[49] Harding K.L., Aguayo V.M., Webb P. (2018). Factors associated with wasting among children under five years old in South Asia: Implications for action. PLoS ONE.

[50] Schoenbuchner S.M., Dolan C., Mwangome M., Hall A., Richard S.A., Wells J.C., Khara T., Sonko B. (2019). The relationship between wasting and stunting: A retrospective cohort analysis of longitudinal data in Gambian children from 1976 to 2016. Am. J. Clin. Nutr..

[51] Development Initiatives (2018). Global Nutrition Report: Shining a Light to Spur Action on Nutrition.

[52] Adams A.M. (1994). Seasonal variations in nutritional risk among children in central Mali. Ecol. Food Nutr..

[53] Stratton M.D., Ehrlich H.Y., Mor S.M., Naumova E.N. (2017). A comparative analysis of three vector-borne diseases across Australia using seasonal and meteorological models. Sci. Rep..

[54] Simpson R.B., Zhou B., Naumova E.N. (2020). Seasonal synchronization of foodborne outbreaks in the United States, 1996–2017. Sci. Rep..

